# Prevalence of influenza vaccination and its association with health conditions and risk factors among Kansas adults in 2013: a cross-sectional study

**DOI:** 10.1186/s12889-016-2884-5

**Published:** 2016-02-24

**Authors:** Jeanie Santaularia, Wei Hou, Ghazala Perveen, Ericka Welsh, Babalola Faseru

**Affiliations:** Kansas Department of Health and Environment, Bureau of Health Promotion, 1000 SW Jackson Suite 200, Topeka, 66619 Kansas USA; Department of Preventive Medicine and Public Health, University of Kansas Medical Center, 3901 Rainbow Boulevard, MS 1008, Kansas City, KS 66160 USA; Department of Family Medicine, University of Kansas Medical Center, 3901 Rainbow Blvd, Kansas City, Kansas 66160 USA

**Keywords:** Influenza, Vaccination, Chronic Disease, Prevention

## Abstract

**Background:**

According to the Centers for Disease Control and Prevention, approximately 5-20 % of people are affected by influenza annually, and influenza causes more than 200,000 hospitalizations each year. The purpose of this study is to estimate the prevalence of influenza vaccination among high risk adults in Kansas.

**Methods:**

The 2013 Kansas BRFSS data (n = 20,712) were analyzed to assess the prevalence of receiving influenza vaccination among Kansas adults, overall and for selected demographic characteristics within the past 12 months. Crude and adjusted prevalence rate ratios were computed using univariate logistic regression models with influenza vaccination as the dependent variable and health conditions or high risk groups as the main independent variables; these models were then adjusted for potential confounding.

**Results:**

Overall, influenza vaccination rate was lower than the Healthy People 2020 target (42.2 % vs. 80 %). The prevalence of receiving influenza vaccination was higher among adults 65 years and older compared to adults 64 years and younger after adjusting for gender, annual household income, education, marital status, insurance status, and race/ethnicity. Similarly, the prevalence of receiving influenza vaccination was higher among adults who have current asthma, or have ever been diagnosed with diabetes, high blood pressure, cancer (excluding skin), and COPD compared to those who did not have these health conditions, as well as pregnant women compared to women who were not pregnant.

**Conclusions:**

Although high risk groups have higher rates of influenza vaccination compared to low risk groups, more concerted efforts are needed to improve seasonal influenza vaccination in Kansas.

## Background

Seasonal influenza, commonly known as “the flu”, is a contagious viral respiratory illness, which can cause mild to severe illness. Unlike the common cold, influenza can lead to hospitalization, life-threatening complications and death [1]. According to the Centers for Disease Control and Prevention (CDC), approximately 5-20 % of people living in the United States are affected by influenza annually, and influenza causes more than 200,000 hospitalizations for influenza related illness and complications each year [1, 2]. Deaths due to influenza vary drastically year to year due to the unpredictability, length and severity of the influenza season. Thus, the range of influenza-related mortality over a thirty-year period, between 1976 and 2007 in the United States, is estimated at a low of approximately 3,000 to a high of approximately 49,000 deaths per year [2]. It is difficult to have a more precise estimate of the annual mortality associated with influenza for several reasons including: influenza is not a reportable disease, influenza is infrequently listed on death certificates [1], and the severity and duration of flu season varies from year to year. Therefore, the influenza specific mortality rates reported annually are only a tip of the iceberg.

The financial burden of influenza in the United States is enormous. One study estimated that annual influenza epidemics result in an average of 610,660 potential life-years lost, 3.1 million hospitalized days, and 31.4 million outpatient visits based on the 2003 U.S. population [3]. The direct medical cost is around $10.4 billion annually, and the projected lost earnings due to influenza related illness and loss of life sums to $16.3 billion annually. The estimated total economic burden of annual influenza epidemics is approximately $87.1 billion [3].

Although adults with certain chronic health conditions and adults in certain high-risk groups are not at increased risk of contracting influenza, they can suffer more serious medical consequences if they contract influenza [3]. For example, adults with chronic health conditions such as asthma are at increased risk of experiencing worsened asthma symptoms when they contract influenza infection [3]. Influenza can also lead to pneumonia and other acute respiratory diseases [4]. One study demonstrated increased mortality of coronary heart disease associated with influenza epidemics [5]. In addition, adults 65 years and older are victims of approximately 90 % of influenza associated deaths [1]. These consequences for people who are at high risk for developing flu-related complications indicate why it is imperative that these adults avoid getting influenza.

Annual vaccination is the most effective way to prevent influenza [6]. Healthy People 2020 set the target prevalence rate for seasonal influenza vaccine for pregnant women and non-institutionalized adults aged 18 to 64 years at 80 % [7]. The target prevalence rate for influenza vaccine for non-institutionalized high-risk adults (adults with chronic health conditions) aged 18 to 64 years and non-institutionalized adults aged 65 years and older was set at 90 % [7]. However, influenza vaccination rates remain below recommendations in the general adult population in the United States. According to CDC, the seasonal influenza vaccine rates for all groups at high-risk of developing influenza-related complications are below the Healthy People 2020 target objective. For the 2010–2011 influenza season, the estimated national coverage was 40.5 % among adults aged 18 years and older. The influenza vaccine coverage was 69.6 % among adults aged 65 years and older and 46.7 % among adults aged 18 to 64 years with at least one selected high-risk chronic health conditions (such as asthma) measured by the Behavioral Risk Factor Surveillance System (BRFSS) [8]. Increasing influenza vaccine uptake is one way to reduce hospitalization and healthcare spending, particularly among the elderly with more than one chronic disease [9].

Vaccines are a cost-effective, core component of any preventive service [10]. Immunization programs utilize surveillance systems to better understand vaccination coverage and identify strategies to reach those at the highest risk for influenza complication. It is critical to monitor influenza vaccination rates among those who are at high risk for developing influenza-related complications in order to avoid the onset of disease, reduce mortality and health care cost since these are the people who have the most serious consequences once infected with influenza.

The purpose of this study is to estimate the prevalence of influenza vaccination among specific high-risk groups susceptible to influenza complications in Kansas in 2013.

## Methods

### Study design and protocol

The Kansas BRFSS is an ongoing, annual, population-based cross-sectional random-digit-dial telephone survey of non-institutionalized adults 18 years and older living in a private residence with landline and/or cell phone service (dual frame sampling) in Kansas. In 2013, the landline component of the dual frame sampling methodology involved disproportionate stratified sampling which included selection of landline telephone numbers within 10 geographic strata. In 2013, the cell phone component of the dual frame sampling methodology comprised of sampling from the entire state as a single stratum. The samples (telephone numbers) that were drawn from each geographic stratum was based on the population size within each geographic stratum.

### Study setting and participants

Kansas is a US Midwestern state. In 2013, the estimated population of Kansas is approximately 2.8 million people with approximately 2.6 million adults 18 years and older. The 2013 Kansas BRFSS survey was administered daily between January 2 and December 31, 2013 in English and Spanish. The study was approved by the Department of Health and Environment Institutional Review Board. A total of 20,712 respondents 18 years and older completed the survey.

### Measures

All of the questions below were asked of all 20,712 respondents 18 years and older. Prevalence of the influenza vaccination was determined by the question: "There are two ways to get the influenza vaccine, one is a shot in the arm and the other is a spray, mist, or drop in the nose called FluMist™. During the past 12 months, have you had either a seasonal flu shot or a seasonal flu vaccine that was sprayed in your nose?". Response options were “yes,” “no,” “don’t know,” and “refused.” Adults who answered “yes” to this question were defined as those who have received the seasonal influenza vaccine.

There were five dichotomous chronic health conditions and five dichotomous high risk groups that were assessed for their association with receipt of influenza vaccination. We examined five health conditions prone to influenza-related complications measured in BRFSS as defined by CDC [11]. Adults were asked individually for each health condition if they had ever been told by a doctor, nurse, or healthcare professional that they had diabetes, high blood pressure, cancer (excluding skin cancer), Chronic Obstructive Pulmonary Disease (COPD), and asthma. Those who indicated that they had been diagnosed with asthma were then asked if they currently had asthma.

The five high-risk groups prone to influenza-related complications, as defined by CDC [11], measured in BRFSS included: people 65 years and older, pregnant women, American Indians, adults living in households with children, and adults with a BMI greater than 40. Adults living in households with children were defined as those who provided a valid numerical response to the question “How many children less than 18 years of age live in your household.” Additionally, respondents’ body weights were estimated by asking, “How much do you weigh without shoes?” and height was measured by, “About how tall are you without shoes?” BMI was then calculated as weight in kilograms divided by height in meters-squared (kg/m^2^). Morbidly obese weight was categorized as body mass index (BMI) equal to or greater than 40 kg/m^2^ calculated using weight and height.

### Statistical analysis

Weighted survey procedures were implemented using SAS software version 9.3 and SAS callable SUDAAN 11.0.1 to account for the complex survey design of BRFSS. A description of weighted survey procedures can be found in the document - “Alternatives for Analysis of Complex Sample Surveys: A Comparison of SAS®, SUDAAN®, and AM® Software” available on-line [12]. Weighted data analysis is important since it also allows the generalization of findings to the whole population, not just those who responded to the survey. The options used in SUDDAAN to do this include: NEST and WEIGHT. The prevalence of receiving influenza vaccination among Kansas adults, along with corresponding 95 % confidence intervals, were calculated overall and for selected demographic characteristics. Univariate logistic regression models were examined with influenza vaccination as the dependent variable and health conditions or high risk groups as the main independent variables. Then, multivariable logistic regression models were examined with influenza vaccination as the dependent variable and health conditions or high risk groups as the main independent variables with additional covariates to adjust for potential confounding. Crude and adjusted prevalence rate ratios (PRR) were computed to examine the prevalence of influenza vaccination among adults with each health condition or each high risk group and significant relationships were determined by 95 % confidence intervals exclusive of 1 and the Rao-Scott Chi-Square test p-value less than 0.05. The key social demographic characteristics that were used to adjust for confounding included age, race/ethnicity, marital status, education, annual household income and healthcare coverage.

## Results

In 2013, the prevalence of receiving influenza vaccination within the past 12 months was 42.2 % (95 % CI: 41.4 - 43.0) among Kansas adults 18 years and older. The prevalence of influenza vaccination by demographic characteristics is shown in Table 1. Prevalence of influenza vaccination status by health risk behaviors and chronic health conditions is demonstrated in Table 2.

**Table 1:** Prevalence of influenza vaccine by socio-demographic characteristics, 2013 KS BRFSS

	Vaccinated Weighted %	95 % CI
Overall	42.2	41.4-43.0
Sex		
Men	37.9	36.7-39.1
Women	46.3	45.2-47.3
Age Groups		
18-24 years	26.5	23.8-29.1
25-34 years	32.9	30.8-35.1
35-44 years	34.8	32.8-36.9
45-54 years	38.8	36.9-40.6
55-64 years	48.6	47.0-50.2
65 + years	64.8	63.6-66.1
Race/ Ethnicity Groups^a^		
White Non-Hispanic	41.6	40.7-42.5
Black Non-Hispanic	37.6	33.3-41.9
Multiracial/Other Race Non-Hispanic	40.4	36.4-44.3
Hispanic	38.1	34.8-41.3
Marital Status		
Married or member of an unmarried couple	46.2	45.1-47.2
Divorced or separated	36.2	34.2-38.3
Widowed	61.5	59.3-63.6
Never married	28.0	26.0-30.0
Education Groups		
Less Than High school	34.0	31.2-36.9
High school or GED	37.7	36.2-39.2
Some College	40.6	39.2-42.0
College Graduate	51.9	50.6-53.2
Annual Household Income Groups		
Less than $15,000	31.7	28.9-34.4
$15,000-$24,999	36.6	34.6-38.7
$25,000-$34,999	40.1	37.7-42.6
$35,000-$49,999	43.0	40.8-45.2
$50,000 or more	47.6	46.3-48.9
Insurance Status		
Uninsured	19.2	17.4-21.1
Insured	46.7	45.9-47.6

^a^Prevalence estimates for race and ethnicity were age-adjusted to the U.S. 2000 standard population

**Table 2:** Prevalence of influenza vaccination status by health risk behaviors and chronic health conditions, Kansas BRFSS 2013

	Percentage of adults		Percentage of adults	
	vaccinated against		not vaccinated	
	influenza^a^		against influenza^a^	
	%	95 % CI	%	95 % CI
Chronic Health Conditions				
Asthma	47.4	44.6-50.2	41.7	40.9-42.5
Diabetes	57.5	55.2-59.9	40.5	39.7-41.4
High Blood Pressure	53.6	52.3-54.9	36.9	35.9-37.9
Cancer	57.8	55.1-60.5	41.0	40.2-41.8
COPD	51.0	48.0-54.0	41.6	40.8-42.4
Health Risk Groups				
Pregnant Women	51.1	42.2-59.9	35.6	33.7-37.5
Household with children	36.6	35.1-38.1	45.4	44.5-46.3
People 65 years or older	64.8	63.6-66.1	36.8	35.8-37.7
People with BMI >40	41.9	38.1-45.6	42.4	41.6-43.3
American Indians	44.5	36.7-52.4	42.3	41.5-43.1

^a^Chronic health condition and health risk group subcategories are not mutually exclusive

Table 3 shows the crude and adjusted prevalence rate ratios of influenza vaccine coverage among Kansas adults with chronic health conditions and in high-risk groups compared to those who do not have the health conditions or are not in the high-risk groups. The prevalence of receiving influenza vaccination within the past 12 months were significantly higher among adults who have current asthma, or have ever been diagnosed with diabetes, high blood pressure, cancer (excluding skin), and COPD compared to those who did not have these health conditions. The prevalence of receiving influenza vaccination within the past 12 months were significantly higher among pregnant women compared to women who were not pregnant; households without children compared to households with children; and adults 65 years and older compared to adults 64 years and younger. After adjusting for gender, annual household income, education, marital status, insurance status, race/ethnicity and age, the prevalence of receiving influenza vaccination within the past 12 months remained significantly higher among adults who have current asthma (PRR: 1.2, 95 % CI: 1.1 – 1.3), or have ever been diagnosed with diabetes (PRR: 1.2, 95 % CI: 1.2 – 1.3), high blood pressure (PRR: 1.2, 95 % CI: 1.2 – 1.3), cancer (excluding skin) (PRR: 1.1, 95 % CI: 1.1 – 1.2), and COPD (PRR: 1.2, 95 % CI: 1.1 – 1.2) compared to those who did not have these health conditions; and pregnant women (PRR: 1.3, 95 % CI: 1.1 – 1.6) compared to women who were not pregnant. After adjusting for gender, annual household income, education, marital status, insurance status, and race/ethnicity, the prevalence of receiving influenza vaccination within the past 12 months remained significantly higher among adults 65 years and older (PRR: 1.6, 95 % CI: 1.5 – 1.6) compared to adults 64 years and younger.

**Table 3:** Crude and adjusted prevalence rate ratios (PRR) of influenza vaccination among health risk behaviors and chronic health conditions, Kansas BRFSS 2013

	Crude			Adjusted		
	PRR	95 % CI	P-Value	PRR	95 % CI	P-Value
Chronic Health Conditions						
Asthma	1.1	1.1-1.2	<.01	1.2	1.1-1.3^a^	<.01
Diabetes	1.4	1.4-1.5	<.01	1.2	1.2-1.3^a^	<.01
High Blood Pressure	1.5	1.4-1.5	<.01	1.2	1.2-1.3^a^	<.01
Cancer	1.4	1.4-1.9	<.01	1.1	1.1-1.2^a^	<.01
COPD	1.2	1.2-1.3	<.01	1.2	1.1-1.2^a^	<.01
Health Risk Groups						
Pregnant Women	1.4	1.2-1.7	<.01	1.3	1.1-1.6^a^	0.03
Household with children	0.8	0.8-0.8	<.01	1.0	1.0-1.1^a^	0.25
People 65 years or older	1.8	1.7-1.8	<.01	1.6	1.5-1.6^b^	<.01
People with BMI >40	1.0	0.9-1.1	0.77	1.1	1.0-1.2^a^	0.14
American Indians	1.1	0.9-1.3	0.57	1.2	1.0-1.4^a^	0.09

^a^Models adjusted for gender, annual household income, education, marital status, insurance status, race/ethnicity, & age

^b^Model adjusted for gender, annual household income, education, marital status, insurance status, & race/ethnicity

## Discussion

The current article is unique in that its findings reflect population-based data of influenza vaccination status and its relation to specific health conditions and groups prone to influenza-related complications in Kansas. In 2013, Kansas’ influenza vaccination prevalence was 42.2 % (95 % CI: 41.4 - 43.0), which is similar to the national prevalence of 41.5 % (95 % CI: 41.4 - 41.9) [13]. Despite this similarity, current influenza vaccine coverage rates remain sub-optimal compared to the Healthy People 2020 target objectives of 80 % vaccination rate among pregnant women and non-institutionalized adults 18 to 64 years; and 90 % vaccination rate for non-institutionalized high-risk adults 18 to 64 years, non-institutionalized adults 65 years and older, and health care personnel [7].

Results from this study show that influenza vaccination increased with age. This positive association is consistent with national estimates [13]. Possible reasons for lower vaccination rates among younger adults may be lack of emphasis on vaccination in the younger population from health care providers and historically low seasonal influenza vaccination coverage in this population [14]. In addition, lower vaccination rates among adults between 18 and 49 years might suggest that younger adults do not feel the need to get vaccinated because they are less prone to severe complications. However, a recent study found that seasonal influenza vaccination reduced the risk of flu-related hospitalization by 71.4 % among adults of all ages [15]. Increasing flu vaccine uptake among this group may not only reduce incident influenza cases but also prevent transmission among the population as a whole, ultimately reducing health care costs. Furthermore, adults 65 years and older have Medicare coverage and may likely have other medical conditions driving their need to access the healthcare system, which can increase their chance of getting the influenza vaccine.

The lowest influenza vaccination prevalence was found among adults without health care coverage (19.2 %) compared to 46.7 % of those with health care coverage (Table 1). In addition, results demonstrated that influenza vaccination rates were lower among sub-populations with lower educational attainment and those with lower annual household incomes. These disparities may reflect poorer access to medical care and/or lower health literacy related to the importance of preventive medical care [16]. It is important to increase flu vaccination rates because immunization is the most effective way to prevent influenza and its complications [6]. Therefore, public health personnel should implement influenza vaccination programs or policies for adults without health care coverage and those with low socioeconomic status.

The prevalence rates of receiving influenza vaccination within the past 12 months were statistically higher among individuals with chronic health conditions prone to influenza-related complications (current asthma, diabetes, high blood pressure, cancer excluding skin, and COPD) compared to those who did not have these conditions. Despite significantly higher seasonal influenza vaccination rates among adults with chronic health conditions, vaccination coverage was low compared to Healthy People 2020 target objectives. Corresponding adjusted prevalence rate ratios remained statistically significant even after adjusting for healthcare coverage and other social demographic factors. This suggests that adults in Kansas with chronic health conditions had statistically higher rates of receiving the influenza vaccine even after taking healthcare coverage and demographic factors into consideration. Despite statistically significant higher prevalence of seasonal influenza vaccination among adults with chronic disease, the prevalence was only 1.1 to 1.2 times higher among those who have the conditions compared to those who do not, after adjustment for social demographic characteristics. This translates to a high number of adults with chronic health conditions not actually getting vaccinated. One potential reason for this may be that adults with chronic health conditions do not consider their health conditions as putting them at high risk for influenza related complications [14].

The prevalence rates of receiving influenza vaccination within the past 12 months were significantly higher among only two health risk groups prone to influenza-related complications: pregnant women and adults 65 years and older. Similar to chronic health conditions, this association likely reflects more routine use of health care and thus more opportunities to receive the vaccination. The prevalence rate of receiving influenza vaccination within the past 12 months was significantly lower among adults who lived in households with children. One potential reason for the lower prevalence among this group could be that adults are less concerned about getting themselves vaccinated as long as the children received the vaccination, since it has been demonstrated that children contribute more to the spreading of influenza in the population [17]. The prevalence rate of seasonal influenza vaccination among pregnant women and adults 65 years and older remained significantly higher even after adjustment. This is especially important since the CDC estimated that more than 200,000 people in the United States are hospitalized each year from flu-related illness, especially adults 65 years of age and older [15].

These findings raise serious concerns about the low levels of influenza vaccination coverage. These results are important and useful in identifying and implementing tailored programs to improve vaccination coverage among adults prone to influenza-related complications. In order to further improve vaccination coverage, providers should address barriers to delivery and acceptance of influenza vaccination among adults with specified health conditions and characteristics.

This study is not without limitations. First, data collected via the BRFSS are cross-sectional; therefore, the demonstrated associations do not infer causal relationships. Second, these findings are based on self-reported data, which may be prone to biases (reporting bias, misclassification bias, etc.). Additional studies examining hospitalizations due to complications of the seasonal influenza, primary care visits due to influenza, and beliefs and attitudes towards immunization could help to further elucidate patterns of seasonal influenza vaccination in Kansas.

## Conclusion

In conclusion, regardless of higher prevalence of influenza vaccination among various subgroups, vaccination remains sub-optimal among persons prone to influenza-related complications. Results from this study can help inform which subgroups may benefit from more intense health education; however, there’s still a need to educate the population as a whole. Some avenues to increase influenza vaccination coverage could include: greater public awareness of the importance of influenza vaccination, especially for the younger population; more public health campaign efforts to target populations at increased risk of influenza-associated complications; stronger recommendations for flu vaccination from professional organizations; increased emphasis on the need for influenza vaccination by healthcare workers; and automated reminder mechanisms for physicians.

## Abbreviations

BRFSS: Behavioral risk factor surveillance system
CDC: Centers for disease prevention and control
COPD: Chronic obstructive pulmonary disease
PRR: Prevalence rate ratios
CI: Confidence intervals
